# Changes in the Antioxidant and Mineral Status of Rabbits After Administration of Dietary Zinc and/or Thyme Extract

**DOI:** 10.3389/fvets.2021.740658

**Published:** 2021-10-22

**Authors:** Katarína Kucková, L'ubomíra Grešáková, Margaréta Takácsová, Anna Kandričáková, L'ubica Chrastinová, Mária Polačiková, Adam Cieslak, Sylwester Ślusarczyk, Klaudia Čobanová

**Affiliations:** ^1^Centre of Biosciences of the Slovak Academy of Sciences, Institute of Animal Physiology, Kosice, Slovakia; ^2^National Agricultural and Food Centre, Research Institute for Animal Production, Luzianky, Slovakia; ^3^Department of Animal Nutrition, Poznan University of Life Sciences, Poznan, Poland; ^4^Department of Pharmaceutical Biology and Botany, Medical University of Wroclaw, Wroclaw, Poland

**Keywords:** organic zinc, thyme, rabbit, antioxidant enzymes, lipid peroxidation, microelement content

## Abstract

This study was aimed at determining the impact of organic zinc (Zn) and thyme extract (TE) administration, given alone or together for 6 weeks, on the antioxidant and mineral status (Zn, Cu, Fe, and Mn) in the plasma and tissues of growing rabbits. A total of 96 rabbits of age 35 days were randomly assigned to one of four treatment groups: a control group (C), a Zn group supplemented with dietary zinc (50 mg/kg), a TE group receiving thyme extract applied in drinking water (1 ml/L), and a Zn + TE group treated with both additives. Lipid peroxidation in the plasma was influenced by Zn intake and in the kidney was affected by both the Zn and TE treatment (*P* < 0.05). Zn supplementation led to a significant increase in glutathione peroxidase activity (*P* = 0.017), total antioxidant capacity (*P* = 0.009) and total thiol groups level (*P* = 0.047) in the kidney, with the highest values occurring in rabbits receiving the combination Zn + TE. Administration of TE influenced Zn content in the kidney (*P* < 0.001), while zinc intake decreased Cu concentration in muscle (*P* = 0.021). In conclusion, the simultaneous administration of organic Zn and TE positively affected the antioxidant response of kidneys and can be used for improving the antioxidant status of growing rabbits.

## Introduction

Dietary zinc and herbal nutraceuticals are known for their antioxidant properties and protective role against oxidative stress in animals (1, 2). Zinc is a redox inactive trace element and an important component of the antioxidant network because it acts as a cofactor of antioxidant enzymes, protects cell membranes against oxidative damage through stabilization of sulfhydryl groups, is involved in the regulation of glutathione metabolism, and is a strong inducer of metallothionein (MT) synthesis, which acts in the sequestration of the reactive oxygen species produced under stress conditions (3–5).

Minerals bound to an organic ligand are considered to be stable in the digestive tract, resulting in their protection from the formation of indigestible complexes with other dietary components; they may thus enable greater mineral absorption (6). Nevertheless, some researchers have reported variable data on zinc bioavailability in poultry (7), pigs (8), or sheep (9), indicating no advantage in the use of organic Zn forms as compared to inorganic forms. The results of our previous experiment carried out on rabbits showed no differences between dietary Zn sources (Zn sulfate, Zn chelate of glycine hydrate, or protein hydrolysate) in tissue deposition and fecal mineral concentration. However, the intake of organic Zn glycinate increased the activity of zinc-containing Cu/Zn superoxide dismutase in the kidney of rabbits fed diets with a Zn dosage above the maximum EU authorized total contents (150 mg Zn/kg complete feed) (10, 11).

The medicinal aromatic herb *Thymus vulgaris* (thyme) which belongs to the Lamiaceae (*Labiatae*) family, originating from the Mediterranean countries in Europe, is a source rich in volatile essential oil and also non-volatile phenolic compounds, mainly phenolic acids and flavonoids (12). There is currently a lot of information about the health benefits of thyme, including antioxidant, antimicrobial, anti-inflammatory, antispasmodic, immunomodulatory, and anti-mutagenic properties, which are associated with its essential oils and chemical components (13–15). Although numerous *in vitro* studies have shown the antioxidant properties of thyme extracts (14, 16), respective experimental *in vivo* evidence is still limited, particularly in the case of rabbits. A series of trials using dietary thyme or its extract have been carried out to study its efficacy in stabilizing lipid oxidation and improving the quality of rabbit meat (17, 18). Placha et al. (19) showed that dietary inclusion of thyme essential oil may improve antioxidant status as well as the intestinal integrity of rabbits. However, no information is available on the interaction between bioactive thyme compounds and zinc regarding their antioxidant efficiency in rabbits.

Based on the advantages mentioned above, we hypothesized that the administration of organic Zn and/or *T. vulgaris* might positively affect the antioxidant status of rabbits after weaning. Thus far, there is a dearth of knowledge about the impact of organic zinc and thyme extract applied alone or in combination on the microelements content in plasma and tissues in growing rabbits. Therefore, the objectives of the present study were to determine the effect of dietary organic Zn supplementation (50 mg/kg diet) and/or thyme extract added to drinking water (1 ml/L water) on the activities of antioxidant enzymes, such as superoxide dismutase (SOD, Cu/Zn SOD), glutathione peroxidase (GPx) and catalase (CAT), lipid peroxidation, total antioxidant capacity and trace elements (Zn, Cu, Fe, and Mn) concentration in the plasma and tissues of rabbits.

## Materials and Methods

The trial was conducted at the rabbit research facility of the National Agricultural and Food Centre, Research Institute for Animal Production, Nitra, Slovak Republic. All procedures were carried out in accordance with the European Community guidelines (EU Directive 2010/63/EU) for the care and use of animals for scientific purposes, and the experimental design was approved by the Local Animal Experimental Ethics Committee and by the Slovak governmental authority (4047/16-221).

### Animal Management and Treatments

Ninety-six weaned rabbits (both sexes, meat lines P91 and M91), 35-days of age (initial body weight 1.1 ± 0.2 kg) were distributed in a completely randomized design into four treatment groups (24 rabbits per group), each consisting of six replicates and four rabbits per replicate. The animals (two rabbits/cage) were housed in standard cages (61 × 34 × 33 cm) in a closed building equipped with a heating and forced ventilation system which allowed the environmental temperature to be adjusted within the range of 22 ± 4°C and the relative humidity varied from 65 to 75% throughout the experiment. The photoperiod was 16L:8D. Rabbits of all treatments were fed with a complete pelleted (pellets of 3.5 mm in average size) basal diet (BD), commonly used in the nutrition of growing rabbits. The ingredients and chemical composition of this BD are represented in Table 1. The feed samples were analyzed in triplicate according to standard procedures (20, 21), as previously described (22).

**Table 1:** Ingredients and chemical composition of the granulated basal diet.

**Ingredients**	**g/kg**	**Analyzed nutrient composition**	**g/kg**
Lucerne meal	360	Dry matter	904
Extracted sunflower meal	55	Crude protein	157
Extracted rapeseed meal	55	Crude fiber	167
Wheat bran	90	Fat	41
Oats	130	Starch	183
Malt sprouts	150	Organic matter	850
Barley grains	80	Acid detergent fiber	225
DDGS^a^	50	Neutral detergent fiber	340
Vitamin and mineral premix^b^	17	Hemicellulose	125
Limestone	10	Cellulose	170
Sodium chloride	3	Ash	83
		Calcium	6.5
		Phosphorus	6.3
		Zinc (mg/kg)	90
		Copper (mg/kg)	20
		Iron (mg/kg)	316
		Manganese (mg/kg)	114
		Metabolic energy (MJ/kg)	10.9

a *DDGS, Dried distiller grains with solubles*.

b *Vitamin/mineral premix provided per kg of complete diet: Vitamin A, 6000 IU; Vitamin D3, 1,000 IU; Vitamin E, 50 mg; Vitamin B1, 1.7 mg; Vitamin B2, 8.0 mg; Vitamin B6, 3.0 mg; Vitamin B12, 0.01 mg; Vitamin K3, 0.5 mg; biotin, 0.2 mg; folic acid, 0.5 mg; nicotinic acid, 70 mg; choline chloride, 700 mg; Mn, 50 mg; Fe, 40 mg; Cu, 30 mg; Se, 0.2 mg*.

The experimental treatments included a control group (C) without additives, a second group (Zn) supplemented with dietary organic zinc, a third group (TE) receiving thyme extract, and a fourth group of rabbits administered a combination of both additives (Zn + TE) over 42 days (35–77 days of age). The appropriate amount of the organic zinc complex Zn proteinate (Bioplex®Zn 15%, Alltech Inc., Nicholasville, KY, USA) was directly mixed with the BD to provide an additional 50 mg Zn/kg. The total analyzed Zn content in the supplemented BD was 146.3 ± 1.8 mg/kg (Zn treatment) or 145.9 ± 1.7 mg/kg (Zn + TE treatment).

The ethanolic extract of thyme (leaves of *Thymus vulgaris* L.) used in our study was a commercial product provided by Calendula a.s., Nová Lubovna, Slovakia, and was applied daily in fresh potable water at a dose of 1 ml/L. For the analysis of the major bioactive compounds, the *T. vulgaris* extract was evaporated to dryness and then 50 mg were dissolved in 3 ml of Milli-Q water (acidified with 0.2% formic acid) and purified by solid-phase extraction using the Oasis HLB 12cc Vac Cartridge (Waters Corp., Milford, USA). The phenolic acids and flavonoids were analyzed by ultra-high-resolution mass spectrometry (UHRMS) using a Dionex UltiMate 3000RS system (Thermo Scientific, Darmstadt, Germany) with a charged aerosol detector connected to a high-resolution quadrupole time-of-flight mass spectrometer (HR/Q-TOF/MS, Impact II, Bruker Daltonic GmbH, Bremen, Germany), as was previously described (23). The amounts of the phenolic acids in thyme extract were calculated as the rosmarinic acid (CAS 537-15-5 (R)-rosmarinic acid) equivalent, and isoquercetin (CAS 482-35-9 quercetin 3-o-glucopyranoside) was used for calculating the amounts of the flavonoids identified. Stock solutions of rosmarinic acid and isoquercetin were prepared in MeOH at concentrations of 3.2 and 4.5 mg/mL, respectively, and kept frozen until used. Calibration curves for these two compounds were constructed based on seven concentration points (from 500 to 3.9 μg/ml). Some of the metabolites identified in *T. vulgaris* extract were previously published (24, 25). The thyme extract consisted of 157.51 mg/g extract of phenolic acids and 135.99 mg/g extract of flavonoids (Table 2). Thymol and carvacrol were quantified by high-performance liquid chromatography analysis according to the modified method of Pisarčíková et al. (26). The concentration of thymol and carvacrol measured in the TE was 0.49 and 0.04 mg/g, respectively. All thyme extract analyses were carried out in triplicate.

**Table 2:** Contents of the flavonoids and phenolic acids (mg/g of extract) identified in the *T. vulgaris* extract.

**RT**	**UV**	***m/z***	**MS^**2**^**	**MS^**2**^**	**Formula**	**Compound**	**Flavonoids**	**Phenolic**	**References**
**(min)**	**(nm)**	**[M-H]^**−**^**		**Fragments**				**acids**	
1.4	284	197.0446	179	135,151,123	C_9_H_10_O_5_	3,4-O-Dimethylgallic acid		0.19	(24), HMDB0041662
3.8	292,319	341.0884	179	135	C_15_H_18_O_9_	1-Caffeoyl-beta-D-glucose		0.655	HMDB0036937
5.2	292,322	305.0706	225	165	C_8_H_18_O_12_	Gallocatechin	7.36		HMDB0038365
6.2	288,320	253.0713	161	179,135	C_12_H_14_O_6_	1-O-Caffeoylglycerol		0.67	102520618^*^
6.4	285,325	387.1675	207	163	C_18_H_28_O_9_	Hydroxyjasmonic acid glucoside		0.69	(24)
7.2	282	609.1483	489	519,369,399	C_20_H_34_O_21_	Flavonoid C-glycosides	0.70		
7.3		371.0994	249	175,121	C_16_H_20_O_10_	Dihydroferulic acid 4-O-glucuronide		0.385	HMDB0041723
8.1	213,272	593.1542	473	353,383,503,575	C_27_H_30_O_15_	Isovitexin 8-C-beta-glucoside	12.10		HMDB0029484
8.2		163.0388	119		C_9_H_8_O_3_	4-Coumaric acid		1.555	HMDB0041592
8.3		507.1882	327	315	C_25_H_32_O_11_	(7'R,8'R)-4,7'-Epoxy-3'-methoxy-4',5,9,9'-lignanetetrol 9'-glucoside		0.205	HMDB0038710
8.5	285,326	375.0731	179	135	C_18_H_16_O_9_	Caffeic acid derivative		2.955	
9.4	282,343	463.0892	301	287	C_21_H_20_O_12_	Isoquercetin	24.225		HMDB0037362
9.6	254,346	447.0945	285		C_21_H_20_O_11_	Kaempferol 7-O-Hex	0.055		(24), HMDB0037572
9.9	284,331	463.0901	287	151,175,135	C_21_H_20_O_12_	Eriodictyol 7-Glucur	0.885		(24)
10.3	262,340	593.1526	285		C_27_H_30_O_15_	Kaempferol 3-O-Hex-Rha	9.18		(24), HMDB0040475
10.5	260,345	447.0948	285		C_21_H_20_O_11_	Luteolin 7-O-Hex	15.69		(24), HMDB0035588
10.6	260,340	461.0731	285		C_21_H_18_O_12_	Luteolin 7-O-Glucur	38.76		(24), HMDB0240541
10.7	285,321	521.1314	323	359,161,197,179,135	C_24_H_26_O_13_	Salviaflaside		29.07	HMDB0033705
11.5	285	625.1210	301	463	C_30_H_26_O_15_	Quercetin 3-(6”-caffeylgalactoside)	5.15		HMDB0037539
11.8	270,328	445.0785	269		C_21_H_18_O_11_	Apigenin-7-Glucur	1.425		HMDB0240480
11.9	290,331	359.0780	161	197,179,135	C_18_H_16_O_8_	Rosmarinic acid		85.34	(24), HMDB0003572
12.0	283,324	567.1730	323	359,161,197,135	C_27_H_36_O_13_	Rosmarinic derivative		0.915	
12.2	287,324	555.1172	161	359,197,135	C_27_H_24_O_13_	Salvianolic acid K		6.98	(24), 10482829^*^
12.7	285,331	475.0902	285	431,372	C_22_H_20_O_12_	Flavonoids	1.83		
13.1	284,330	609.1272	285	447	C_30_H_26_O_14_	Rutin	1.41		HMDB0003249
13.7	287,323	569.1323	161	339,193,359,137	C_28_H_26_O_13_	Rosmarinic derivative		19.90	
14.1	287,323	493.1157	161	359,197,135	C_26_H_22_O_10_	Salvianolic acid A		2.855	5281793^*^
14.3	341	285.0413			C_15_H_10_O_6_	Luteolin	3.08		(25), HMDB0005800
14.5	287,330	537.1061	331	197,161,135	C_27_H_22_O_12_	3'-O-(8”-Z-caffeoyl) rosmarinic acid		0.395	(24)
14.9	285,328	521.1468	341	197,297,179,135	C_28_H_26_O_10_	Rosmarinic derivative		2.22	
15.9	285,329	387.1458	161	179,135,327	C_21_H_24_O_7_	Rosmarinic derivative		0.275	
16.0	285	269.0454	151		C_15_H_10_O_5_	3',4',5-Trihydroxyflavone	0.255		HMDB0034004
18.2	281,329	537.1425	179	359,135,161	C_28_H_26_O_11_	Lithospermic acid A		2.255	6441498^*^
18.6	278,337	343.0829	328	313,298,285	C_18_H_16_O_7_	Dihydroxy-trimethoxyflavone	4.52		(25), HMDB0040321
19.2	284,328	343.0833	313	328,298	C_18_H_16_O_7_	5,7-Dihydroxy-3',4',6-trimethoxyflavone	2.935		(25), HMDB0029469
19.6	283,333	373.0931	343	358,328	C_19_H_18_O_8_	4',5-Dihydroxy-3',6,7,8-tetramethoxyflavone	6.43		(25), 181092^*^
21.5	216,282	329.1773	286		C_20_H_26_O_4_	Carnosol			HMDB0002121
**Total flavonoids and phenolic acids**							**135.99**	**157.51**	

*RT, retention time; MS^2^, main ion; Hex—hexosyl (glucosyl, galactosyl); Rha—(rhamnosyl); Glu—(glucosyl); Glucur—(glucuronyl, galacturonyl); HMDB ID—The Human Metabolome Database; m/z, mass-to-charge ratio*.

* *PubChem ID (based on MetFrag Online DataBase), and supported by the literature (24, 25)*.

The granulated diets and drinking water were available *ad libitum* throughout the whole experiment. Weekly measurements (from 35 to 77 days of age) of body weight and feed consumption for each replicate of the treatments (24 rabbits/treatment) were taken to calculate the average daily feed intake, average daily weight gain, and feed conversion ratio. At 77 days of age, all rabbits were weighed individually and the final average body weight was calculated for each treatment. Mortality within each replicate was monitored daily throughout the experiment and data pertaining to any dead animal were excluded from the calculation.

### Blood and Tissue Sampling

At the end of the trial, blood samples were taken from the peripheral ear vein of the randomly selected starved animals from each group (six animals/treatment, one male from each replicate), and the rabbits were subsequently slaughtered (at the abattoir of the National Agricultural and Food Centre, Nitra-Luzianky, Slovakia) for tissue sample collection. Animals were electrical stunned and immediately bled by cutting the jugular veins and the carotid arteries. Heparinized blood was centrifuged at 1,180 × g for 15 min. to obtain plasma samples. The liver, kidney, jejunum, and muscle (*musculus longissimus dorsi*) tissues were quickly excised and flushed with saline solution. Sampling was done from the identical area of each relevant tissue, the jejunal mucosa was scraped off with glass slides on ice and all samples were stored at −80°C until further analysis.

### Antioxidant Status Assessment

Determination of antioxidant enzyme activities, lipid peroxidation, and total antioxidant capacity of plasma and tissues has already been described in more detail by Cobanová et al. (1). We performed a duplicate analysis of the same sample, which has been independently prepared and analyzed for the same parameters to monitor and assess the precision of the analytical method. The activity of total superoxide dismutase (SOD) in tissues was measured using the spectrophotometric method based on pyrogallol autoxidation following the method of Marklund and Marklund (27). For each sample, a parallel determination was done in the presence of 1 mM KCN under the same conditions, and the activity of Cu/Zn SOD was calculated as the activity inhibited by KCN. The activity of blood glutathione peroxidase (GPx) was evaluated spectrophotometrically using a commercial Ransel kit (Randox Laboratories, Ltd., London, UK). Enzymatic activity was monitored at a wavelength of 340 nm for 3 min, and the results are expressed in units (U) per g of hemoglobin (Hb). The blood Hb content was analyzed using a commercial kit (Randox Laboratories, Ltd., London, UK), and a calibration curve was prepared with a set of cyanmethemoglobin standards following the manufacturer's instructions. The activity of GPx in tissues was measured by monitoring the oxidation of NADPH at 340 nm according to Paglia and Valentine (28). One unit of GPx activity was defined as the amount of sample that catalyzed the oxidation of 1 μmol NADPH per min under assay conditions. The activity of catalase (CAT) in tissues was determined following the method of Aebi (29), which involves monitoring the disappearance of H_2_O_2_ in the presence of tissue homogenate at 240 nm. One unit of CAT activity was defined as the amount of enzyme needed to decompose 1 μmol H_2_O_2_ per min under assay conditions. The total antioxidant capacity (TAC) of plasma and tissues was assessed using a ferric reducing antioxidant power (FRAP) assay (30). A ferrous sulfate solution was used to create a standard curve, and the results were expressed in mmol Fe^2+^ formed per liter of plasma or μmol Fe^2+^ per g of tissue protein. The enzymatic assays and spectrophotometric determination of TAC in plasma and tissues were measured using a UV/visible Spectrophotometer (Shimadzu UV-2550, Kyoto, Japan). All reagents for the analysis of antioxidant parameters were of the highest grade commercially available and were purchased from Sigma-Aldrich (Steinheim, Germany), Merck (Darmstadt, Germany), AppliChem GmbH (Dermstadt, Germany) and Lach:Ner (Czech Republic). Estimation of lipid peroxidation was done by measuring plasma and tissues malondialdehyde (MDA) concentration by the fluorometric thiobarbituric acid-reactive substances method, as described by Jo and Ahn (31) using a fluorometer (Shimadzu RF-1501, Kyoto, Japan). The standard curve of 1,1,3,3-tetramethoxypropane (Sigma-Aldrich) was used as the MDA precursor. Protein concentrations in the tissues were determined according to Bradford assay (32) in microtitration plates using bovine serum albumin as a standard and measured on a microplate reader (Apollo 11 LB913, Berthold Technologies GmHB & Co. KG, Bad Wildbad, Germany). The Ellman (33) method was used to assess total sulfhydryl (SH) or thiol groups content in plasma and tissues based on the reaction of 5,5′-dithio-bis (2-nitrobenzoic acid) with protein thiol groups measured at 412 nm (UV/VIS Spectrophotometer, Shimadzu UV-2550, Kyoto, Japan). The concentration of the SH groups was calculated using reduced glutathione as the standard, and the results are expressed in mmol per liter of plasma or μmol per g of tissue. Plasma metallothionein (MT) level was determined using a commercial ELISA kit (Rabbit ZnMT, BlueGene Biotech, Shanghai, China) following the manufacturer's instructions. The optical density of the samples was measured at 450 nm using a microplate reader (Apollo 11 LB913, Berthold Technologies GmHB & Co. KG, Bad Wildbad, Germany).

### Trace Mineral Analysis

Mineral (Zn, Cu, Fe, and Mn) content in the diets, plasma, and tissues were analyzed according to a previously reported method (1, 34). Dried, ground samples (except plasma) were acid digested prior to mineral analysis using microwave-assisted digestion in an MWS 4 Speedwave device (Berghof Co., Eningen, Germany). The concentrations of Zn, Cu, Fe, and Mn were measured by flame atomic absorption spectrometry using a double-beam atomic absorption spectrometer (AAS, AA-7000 Series, Shimadzu Co., Kyoto, Japan). The Mn level in plasma and muscle was determined using a graphite furnace AAS (GFA-7000, Shimadzu Co., Kyoto, Japan) with deuterium background correction and pyrolytic-coated graphite tubes. To verify instrument accuracy a particular certificate reference material was included in each analysis (34). The analyzed mineral concentrations in the certified reference material are presented as Supplementary Material. All samples were analyzed in duplicate.

### Statistical Analysis

All statistical analyses were performing using the GraphPad Prism software (GraphPad Prism version 8.4.2., GraphPad Software, San Diego, CA, USA), and the differences between treatments were evaluated by two-way analysis of variance (ANOVA). Experimental data were analyzed as a 2 × 2 factorial in a randomized complete block design representing two main factors: zinc (with and without) and thyme extract (with and without), followed by Tukey's *post–hoc* test for pairwise multiple comparisons, where appropriate. Differences at *P* < 0.05 were considered significant. The Least Significant Difference test (Fisher's LSD) was applied *post–hoc* to determine significant differences among the treatments in the case of significant interaction (Zn × TE). The association between the antioxidant variables was evaluated using Pearson's correlation coefficient. The data presented are the mean values and pooled standard errors of the mean (SEM).

## Results

### Growth Performance

All animals appeared healthy; three animals (one/group C and two/group TE) died during the course of the experiment. Application of zinc and/or thyme extract did not significantly influence the growth performance of the rabbits. The average daily body weight gain was in the range of 38.7–41.5 g/d, and feed conversion ratios were within the range of 3.0–3.5 g/g. The average final body weight (mean ± SEM) of the rabbits at 77 days of age was 2859 ± 114, 2830 ± 49, 2792 ± 61, and 2786 ± 37 g for Zn + TE, Zn, TE, and C groups, respectively. These results were expected for this age of growing rabbits, indicating that growth performance was adequate.

### Antioxidant Status

As shown in Table 3, there were no significant effects of Zn and TE treatment or interaction between additives on blood GPx activity, TAC, MT, and SH level in the plasma. The plasma lipid peroxidation was significantly affected by Zn supplementation (*P* = 0.032), with decreased MDA values occurring in the Zn + TE treatment compared to rabbits receiving the TE only.

**Table 3:** The effect of organic zinc and/or thyme extract administration on the antioxidant indices and microelements concentration in plasma or blood of rabbits.

**Item**	**Treatment**				**SEM**	***P*** **-value**		
	**C**	**Zn**	**TE**	**Zn + TE**		**Zn**	**TE**	**Zn × TE**
Blood GPx (U/g Hb)	124.7	148.4	131.8	125.2	5.048	0.402	0.429	0.143
**Plasma**								
TAC (mmol/L)	0.547	0.670	0.560	0.572	0.024	0.170	0.380	0.252
SH (mmol/L)	0.292	0.299	0.304	0.308	0.010	0.818	0.622	0.953
MDA (μmol/L)	0.188^a,b^	0.182^a,b^	0.198^b^	0.146^a^	0.007	0.032	0.312	0.083
MT (μg/L)	16.13	13.28	15.40	12.90	0.922	0.171	0.772	0.927
Zinc (mg/L)	1.60	1.49	1.67	1.45	0.045	0.077	0.836	0.525
Copper (mg/L)	0.788	0.687	0.808	0.655	0.032	0.056	0.929	0.682
Iron (mg/L)	2.05	2.03	2.05	1.97	0.121	0.860	0.904	0.914
Manganese (μg/L)	6.78	6.43	7.08	7.13	0.199	0.720	0.229	0.629

*C, control; Zn, zinc proteinate; TE, thyme extract; GPx, glutathione peroxidase; Hb, hemoglobin; TAC, total antioxidant capacity; SH, sulfhydryl groups; MDA, malondialdehyde; MT, metallothionein*.

a,b *Means with different superscript letters in a row are significantly different (P < 0.05) using Tukey's post-hoc test*.

Alterations of antioxidant parameters in tissues are presented in Table 4. Neither the total SOD nor Cu/Zn SOD activity in tissues was significantly affected by the treatment. The receiving of dietary Zn significantly increased GPx activity in the kidney (*P* = 0.017), with the highest activity recorded in the group treated with the Zn and TE combination compared to the control group, while the liver tissue showed only a tendency toward higher GPx values (*P* = 0.060) due to Zn treatment. The activity of CAT in the liver and kidney tissues was not significantly influenced by the treatment.

**Table 4:** The effect of organic zinc and/or thyme extract administration on the antioxidant enzyme activities, total antioxidant capacity, total thiols level, and lipid peroxidation in the tissues of rabbits.

**Item**	**Treatment**				**SEM**	***P*** **-value**		
	**C**	**Zn**	**TE**	**Zn + TE**		**Zn**	**TE**	**Zn × TE**
**Total SOD (U/mg protein)**								
Liver	51.35	47.37	52.26	54.54	1.674	0.805	0.249	0.368
Kidney	65.90	60.11	68.34	63.67	2.580	0.341	0.583	0.918
Jejunal mucosa	15.68	17.68	18.71	16.65	0.837	0.987	0.566	0.253
**Cu/Zn SOD (U/mg protein)**								
Liver	40.62	34.71	38.12	45.14	1.705	0.866	0.235	0.060
Kidney	46.84	41.69	46.76	44.88	2.313	0.479	0.753	0.741
Jejunal mucosa	12.68	14.93	15.38	13.85	0.783	0.826	0.620	0.255
**GPx (U/g protein)**								
Liver	15.02	18.05	14.46	16.25	0.632	0.060	0.338	0.612
Kidney	18.00^a^	22.42^a,b^	21.29^a,b^	25.54^b^	0.958	0.017	0.069	0.960
**CAT (U/mg protein)**								
Liver	46.17	48.04	48.99	52.96	1.571	0.370	0.238	0.744
Kidney	54.07	49.90	53.53	66.12	2.438	0.357	0.094	0.075
**TAC (μmol/g protein)**								
Liver	37.71	38.48	39.57	40.38	0.998	0.708	0.380	0.992
Kidney	23.70^a^	26.50^a,b^	24.56^a,b^	28.88^b^	0.708	0.009	0.202	0.547
**SH (μmol/g tissue)**								
Liver	17.25	15.44	16.75	18.00	0.456	0.753	0.256	0.099
Kidney	13.50	13.71	12.77	15.10	0.330	0.047	0.588	0.094
**MDA (nmol/g protein)**								
Liver	50.32^B^	39.95^A,B^	33.35^A^	48.93^B^	2.505	0.561	0.374	0.008
Kidney	83.77^b^	71.01^a,b^	70.41^a,b^	64.10^a^	2.561	0.045	0.035	0.478
Muscle	11.35	11.82	10.56	10.81	0.442	0.698	0.342	0.903

*C, control; Zn, zinc proteinate; TE, thyme extract; SOD, total superoxide dismutase; Cu/Zn SOD, Cu/Zn superoxide dismutase; GPx, glutathione peroxidase; CAT, catalase; TAC, total antioxidant capacity; SH, sulfhydryl groups; MDA, malondialdehyde*.

a,b *Means with different superscript letters in a row are significantly different (P < 0.05) using Tukey's post-hoc test*.

A,B *Means with different superscript letters in a row are significantly different (P < 0.05) using Fisher's Least Significant Difference (LSD) post-hoc test*.

Intake of Zn significantly influenced the antioxidant capacity of the kidney (*P* = 0.009), with the highest values found in rabbits treated Zn and TE together compared to the control group. No significant effect on the liver TAC was recorded due to treatment. A similar pattern was observed in the total SH group content, with the significantly higher values in the kidney of rabbits receiving Zn (*P* = 0.047), while no effect of treatment was detected in the liver. An interaction of Zn × TE was identified for lipid peroxidation in the liver (*P* = 0.008), with decreased MDA concentration when TE was administered alone compared to control rabbits and those receiving the combination of both additives. Lipid peroxidation in the kidney was significantly affected by both the zinc (*P* = 0.045) and the thyme (*P* = 0.035) treatment, with the lowest MDA values detected in rabbits receiving the combination of Zn and TE compared to the control group (*P* < 0.05). No significant effect on MDA concentration in muscle was observed due to treatment (Table 4).

**Figure 1:**
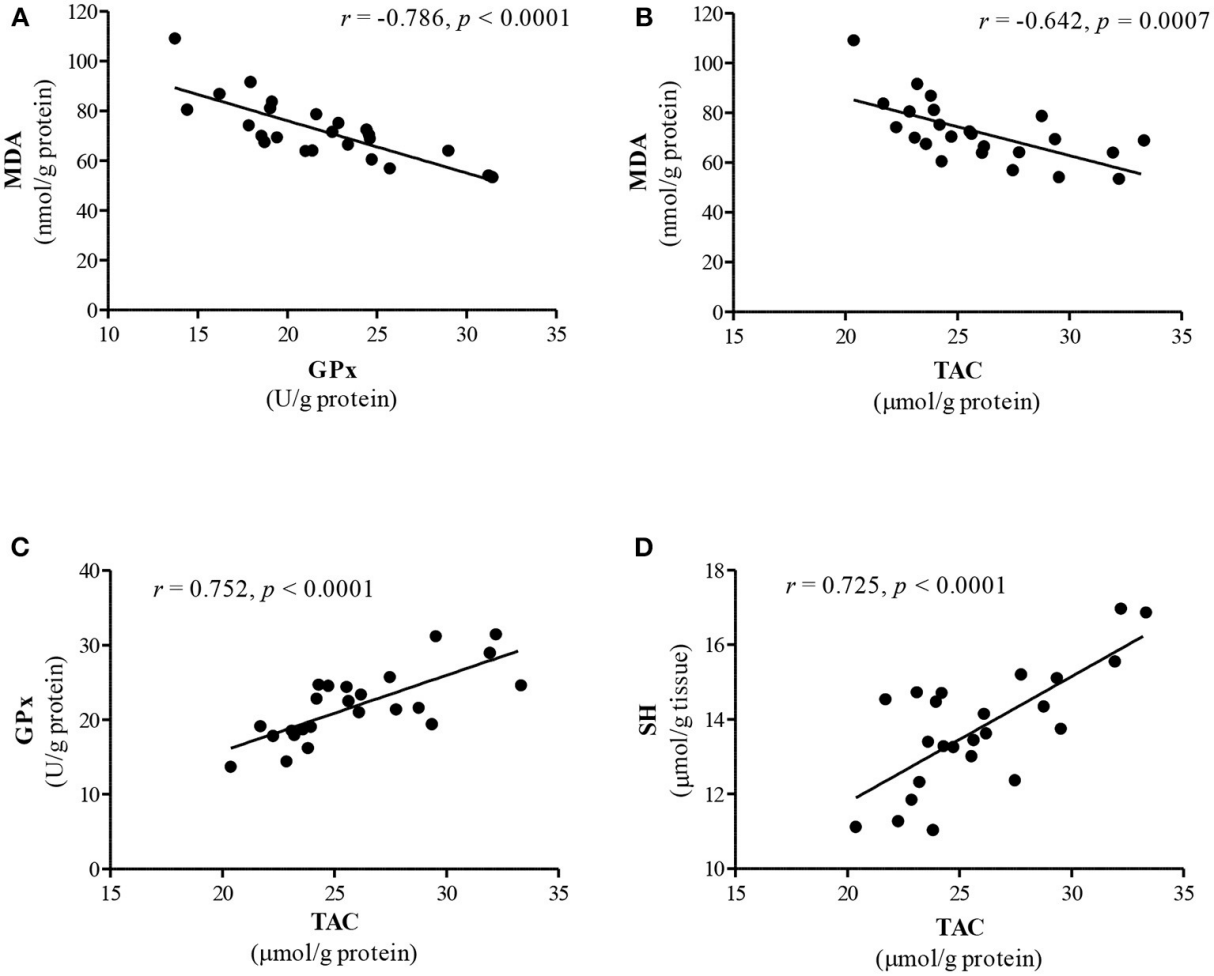
The interrelationship between lipid peroxidation and antioxidant parameters in the kidney. **(A)** MDA level vs. GPx activity; **(B)** MDA level vs. TAC; **(C)** TAC vs. GPx activity; **(D)** TAC vs. SH groups. Data analyzed using Pearson's correlation test and presented as a correlation coefficient (*r*).

The relationship between antioxidant parameters and lipid peroxidation in renal tissue showed a negative significant correlation between MDA concentration and GPx activity (*r* = −0.786, *P* < 0.0001, Figure 1A), TAC (*r* = −0.642, *P* = 0.0007, Figure 1B) and total SH groups content (*r* = −0.514, *P* = 0.010). A strong positive correlation was observed between TAC and GPx activity (*r* = 0.752, *P* < 0.0001, Figure 1C) and total SH groups content (*r* = 0.725, *P* < 0.0001, Figure 1D). The activity of GPx also positively correlated with SH content (*r* = 0.565, *P* = 0.004).

### Mineral Profile

The concentration of microelements in plasma (Table 3) was not influenced by the Zn and TE treatment, but a tendency toward a decreased Cu level (*P* = 0.056) in rabbits treated with Zn was determined.

The concentrations of microelements (Zn, Cu, Fe, and Mn) in tissues are presented in Table 5. The administration of thyme extract to rabbits significantly affected Zn concentration in the kidney (*P* = 0.0004), and the highest values were found in the group receiving Zn alone compared to those treated with TE or the combination TE + Zn. No significant effects on the Zn level in the liver, jejunal mucosa, and muscle were observed due to treatment. There was no effect of Zn and/or thyme treatment on the Cu level in the liver, kidney, and jejunal mucosa, while the intake of organic Zn significantly decreased the Cu concentration in muscle (*P* = 0.021). No significant effects on Mn and Fe tissue deposition were detected due to treatment.

**Table 5:** The effect of organic zinc and/or thyme extract administration on the concentration of microelements (Zn, Cu, Fe, and Mn) in the tissues of rabbits.

**Item**	**Treatment**				**SEM**	***P*** **-value**		
	**C**	**Zn**	**TE**	**Zn + TE**		**Zn**	**TE**	**Zn × TE**
**Zinc (mg/kg DM)**								
Liver	121.6	127.2	123.2	122.0	3.170	0.751	0.793	0.624
Kidney	120.4^a,b^	123.2^b^	110.3^a^	110.6^a^	1.731	0.573	0.0004	0.645
Muscle	20.10	20.50	21.57	22.48	0.439	0.448	0.060	0.764
Jejunal mucosa	135.5	126.8	118.9	122.3	3.946	0.744	0.206	0.465
**Copper (mg/kg DM)**								
Liver	37.90	43.97	31.89	43.92	2.665	0.105	0.575	0.581
Kidney	17.96	17.49	17.38	18.24	0.198	0.622	0.830	0.107
Muscle	0.967	0.867	1.147	0.877	0.042	0.021	0.214	0.264
Jejunal mucosa	9.29	7.55	9.69	9.37	0.382	0.172	0.144	0.339
**Iron (mg/kg DM)**								
Liver	282.4	252.2	266.1	274.2	9.094	0.563	0.881	0.321
Kidney	172.7	185.9	177.2	179.0	3.004	0.231	0.841	0.362
Muscle	16.38	16.18	14.79	15.05	0.442	0.975	0.145	0.803
Jejunal mucosa	128.2	124.1	145.7	117.9	6.666	0.266	0.690	0.408
**Manganese (mg/kg DM)**								
Liver	5.57	5.80	5.49	5.13	0.195	0.867	0.358	0.471
Kidney	7.24	7.42	7.65	8.39	0.263	0.394	0.210	0.602
Muscle	0.372	0.363	0.390	0.396	0.015	0.948	0.446	0.824
Jejunal mucosa	11.74	10.42	13.38	10.70	0.533	0.064	0.359	0.515

*C, control; Zn, zinc proteinate; TE, thyme extract; DM, dry matter*.

a,b *Means with different superscript letters in a row are significantly different (P < 0.05) using Tukey's post-hoc test*.

## Discussion

### Growth Performance

Despite the thyme effect on stimulation of appetite and feed intake as well as its ability to enhance nutrients utilization in growing rabbits (14, 35), no effect of thyme extract administration on growth performance was observed in our trial. Treatment with Zn and TE alone or in combination did not significantly affect the rabbits' final body weight, average daily weight gain, and feed conversion ratio. Similarly, our previous results did not show any significant effect of zinc supplementation (100 mg Zn/kg) from inorganic or organic sources on the growth performance of rabbits (36). Our results are in accordance with those of Gerencsér et al. (37), who did not observe any substantial effect of thyme dietary supplementation (3%) on feed intake, daily weight gain, and health status of rabbits. In contrast, treatment with thyme aqueous extract (50 mg/kg b.w.) or dietary thyme essential oil resulted in a significant increase in daily body weight gain of rabbits and improved feed conversion ratio compared to control animals (38, 39).

### Antioxidant Status

Our previous study showed that dietary Zn supplementation at the level of 100 mg/kg significantly elevated Cu/Zn SOD activity in the kidney of rabbits receiving Zn glycinate, but no significant effect of Zn proteinate was detected (11). Despite the fact that Zn is a structural component of the cytosolic Cu/Zn SOD, zinc supplementation did not influence the activity of this antioxidant enzyme in our study, which can be ascribed to the unchanged content of Zn and/or Cu content in rabbits' tissues after treatment. However, the antioxidant effect of Zn supplementation was reflected in increased activity of GPx, TAC, and total SH content in the kidney tissue along with decreased MDA level in this tissue (Table 4) and plasma (Table 3), as well. Considering that Zn induces the synthesis of proteins rich in sulfhydryl groups, in particular glutathione (the principal intracellular non-protein thiol) and metallothionein (MT, cysteine-rich protein) (5), this fact could be the reason for a significant increase in the total SH group concentration in the kidney and can lead to a consequent reduction in lipid peroxidation. In addition, zinc is capable of protecting protein sulfhydryl groups from oxidation and reducing their reactivity due to its direct binding to the sulfhydryl (3). Zinc is directly involved in glutathione *de novo* synthesis by regulating the expression of glutamate-cysteine ligase and in this way indirectly affects the activity of GPx (5), which can be an explanation for the higher GPx activity in our study.

Zinc is a strong inducer of MT synthesis in renal proximal tubular cells *in vitro* on the RNA as well as on the protein level, and its antioxidant effect is comparable to that of glutathione (40), suggesting that MT plays a major protective role in the kidney and its function, which is possibly influenced by Zn administration (41). In the current experiment, we measured only plasma MT level, and no significant differences were detected among treatments; the total SH content of plasma also followed a similar pattern (Table 3). However, MT occurs in a low concentration in the plasma under normal physiological conditions, because of its main intracellular location. It appears that only in the case of stress or the administration of certain metals would it be appropriate to use the plasma MT level as a marker reflecting changes in the intracellular MT synthesis (42).

Our correlation study indicates that there is a negative significant correlation between MDA level and GPx activity (Figure 1A) as well as TAC (Figure 1B) in the kidney. This finding supports the concept of a reduction of lipid peroxidation by activation of antioxidant defense in the renal tissue. However, it should be stressed that rabbits treated simultaneously with Zn and TE exhibited the highest improvement in antioxidant parameters in the kidneys. This finding probably indicates that the synergistic action of both additives is involved in improving the renal antioxidant defense in growing rabbits. Ratliff et al. (43) reported that dietary antioxidants and naturally occurring phenolic compounds may influence renal function because of their ability to enhance the endogenous antioxidant defense system through scavenging free radicals and stimulation of various antioxidants, including glutathione, thioredoxin, SOD and catalase, *via* NrF-2 (nuclear factor erythroid 2-related factor) upregulation. The renal antioxidant system protects the kidney against oxidative damage in every nephron segment, including renal vessels, glomeruli and tubules, and subsequently maintains normal kidney function (43). The current results further support our previous findings that organic Zn dietary supplementation may improve the antioxidant status in the kidney of rabbits (11).

The antioxidant properties of thyme have been demonstrated through various *in vitro* methods as well as *in vivo* using thyme essential oil, extract, or leaves in traditional treatment or animal nutrition (14, 19, 44, 45). A positive effect of dietary supplementation of rabbits with thyme oil (0.5 g/kg DM) on increasing the total antioxidant status of plasma and GPx activity in the liver as well as reducing the malondialdehyde level in the duodenal mucosa has been reported (19). In our study, administration of thyme extract decreased lipid peroxidation in the kidney and liver tissues, and the combined treatment with Zn resulted in increased GPx activity and TAC of the kidney (Table 4), as was mentioned above. It has been well documented that the antioxidant effect of thyme can be primarily attributed to the high content of phenolic constituents and their ability to neutralize free radicals (46). The antioxidant activity of phenolic compounds contained in thyme extract may be related to their redox properties; they act as a hydrogen donor to free radicals and also possess the metal-chelating ability to protect lipids of biological membranes against oxidation (13). In general, thymol and its isomer carvacrol have been reported to be the major components of thyme essential oil and showed a beneficial effect on antioxidant enzyme activities and inhibition of lipid oxidation *in vivo* (47). Rosmarinic acid is the most abundant polyphenolic compound in thyme extract, and it has been recognized for strong antioxidant activity as well as the flavonoids luteolin and quercetin (16, 44, 45, 48) determined in a lower quantity in the TE used in our trial (Table 2). In this context, the antioxidant effectiveness of the thyme plant and its extracts is based on the high number of various bioactive compounds with potential antioxidant activity that probably act synergistically.

### Mineral Profile

In the current study, dietary Zn supplementation by the amount of 50 mg/kg did not affect Zn levels in plasma and tissues, while administration of thyme decreased Zn concentration in kidney tissue compared to rabbits receiving Zn alone (Table 5). These results are partially similar to our previous finding (11), when dietary intake of Zn proteinate (100 mg Zn/kg) did not influence plasma, kidney, and muscle Zn concentration but resulted in higher liver Zn content. The results of Yan et al. (49) showed increased Zn concentrations in the liver of growing rabbits receiving dietary organic Zn sources (Zn methionine, Zn glycine, or Zn lactate) at the dose of 80 mg Zn/kg for 49 d. Although organic forms of Zn are considered to be more bioavailable compared to the inorganic salts used in animal nutrition, discrepancies in the relative bioavailability have also been observed among various commercial organic mineral sources, which might be attributed to their different chemical characteristics and the degree of chelation or complexation of the Zn ion to organic ligands (50, 51). The weak chelation strength of Zn proteinate observed by Liu et al. (51) may be an explanation for the non-response in plasma and tissue Zn concentration determined in our experiment. It is important to note that circulating zinc concentration plays an important role in maintaining whole-body zinc homeostasis, and the plasma or serum Zn level is unrelated to zinc intake (52). This could also be the reason why plasma zinc did not change after 6 weeks of zinc administration to rabbits in our experiment.

Several factors that can interfere with microminerals absorption are known (53). It has been reported that a reduction in Cu status is observed unless the diet contains more Zn than is recommended, and this can be explained on the basis of interference in Cu absorption by Zn at the intestinal level (54). In most cases, excess dietary Zn induces synthesis of metallothionein within enterocytes, which binds Cu (and Zn) and subsequently is excreted with the feces when the enterocytes are sloughed off (6). In the current study, no statistically significant difference among treatments was observed regarding the Cu concentration in the liver, kidney, and jejunal mucosa; however, receiving diets with Zn dosages up to the maximum EU-authorized total content in the complete feed (150 mg Zn/kg) tended to decrease the Cu level in the plasma. Even though muscle is considered a tissue with less trace mineral deposition, making small changes difficult to detect (55), we found a significantly lower Cu level in muscle (*m. longissimus dorsi*) due to Zn intake (Table 5). This finding is not in line with our previous results, which showed that feed supplementation with organic Zn proteinate (100 mg Zn/kg) did not interfere with Cu absorption in the duodenal mucosa, resulting in significantly higher Cu accumulation in the liver, while no response was noted in plasma and muscle of rabbits.

It has been reported that plant extracts may directly or indirectly act as a factor that elevates bioavailability of minerals from the diet. This most likely occurs due to fiber fermentation as well as bacterial production of short-chain fatty acids, and a subsequent decrease in pH may increase mineral solubility and subsequently their absorption (56). By contrast, phenolic acids and flavonoids have been shown to reduce the absorption of trace elements, such as iron, zinc, and copper, probably as a consequence of chelation by galloyl and catechol groups (57). In our study, the plasma and tissue concentration of Zn, Cu, Fe, and Mn was not affected by thyme administration, indicating that thyme extract in the concentration of 1 ml/L had no substantial effect on microelements uptake in the tissues of rabbits. However, Zn concentration in the kidney was influenced by TE administration and was significantly decreased in rabbits treated with TE alone or in combination with Zn compared to those receiving the zinc only. Further research is required to elucidate the effect of the thyme bioactive compounds on the transport system of minerals in the gut of rabbits.

## Conclusions

The results of this study indicate that organic zinc and thyme, administered both alone or in combination, may positively affect tissue antioxidative status, leading to a reduction in lipid peroxidation, and that they can be used as an efficacious source of exogenous antioxidants for growing rabbits. Overall, the simultaneous treatment with organic Zn and thyme extract was shown to be most effective in protecting kidney tissue from oxidative damage. Our results demonstrate that the administration of thyme extract in drinking water did not significantly influence tissue mineral uptake, while dietary organic zinc supplementation resulted in a decrease in Cu concentration in muscle. This study helps to clarify the interaction between thyme bioactive compounds and microelements absorption in order to improve their bioavailability and antioxidant effect in animals; however, further research is needed to determine the optimal dosage and source of additives used to be more efficacious.

## Data Availability Statement

The original contributions presented in the study are included in the article/Supplementary Material, further inquiries can be directed to the corresponding author.

## Ethics Statement

The animal study was reviewed and approved by Ethical Committee of the Institute of Animal Physiology, Centre of Biosciences of SAS, and by the State Veterinary and Food Office of the Slovak Republic (resolution number Ro-4047/16-221).

## Author Contributions

KK, MT, and AK formal analysis and investigation. SŚ bioactive compound analysis. L'G mineral analysis. AC and MP data curation. L'C project administration and supervision. KČ conceptualization, funding acquisition, writing—review, and editing. All authors have read and approved the finalmanuscript.

## Funding

This research was funded by the Slovak Research and Development Agency under contract number APVV-17-0297.

## Conflict of Interest

The authors declare that the research was conducted in the absence of any commercial or financial relationships that could be construed as a potential conflict of interest.

## Publisher's Note

All claims expressed in this article are solely those of the authors and do not necessarily represent those of their affiliated organizations, or those of the publisher, the editors and the reviewers. Any product that may be evaluated in this article, or claim that may be made by its manufacturer, is not guaranteed or endorsed by the publisher.

## References

[1] Cobanová K Váradyová Z Grešáková L Kucková K Mravčáková D Várady M. Does herbal and/or zinc dietary supplementation improve the antioxidant and mineral status of lambs with parasite infection? Antioxidants. (2020) 9:1172. 10.3390/antiox9121172 33255492 PMC7761366

[2] Serra V Salvatori G Pastorelli G. Dietary polyphenol supplementation in food producing animals: effects on the quality of derived products. Animals. (2021) 11:401. 10.3390/ani11020401 33562524 PMC7914517

[3] Powell SR. The antioxidant properties of zinc. J Nutr. (2000) 130:1447S−54S. 10.1093/jn/130.5.1447S 10801958

[4] Oteiza PI. Zinc and the modulation of redox homeostasis. Free Radic Biol Med. (2012) 53:1748–59. 10.1016/j.freeradbiomed.2012.08.568 22960578 PMC3506432

[5] Marreiro DN Cruz KJC Morais JBS Beserra JB Severo JS Oliveira ARS. Zinc and oxidative stress: current mechanisms. Antioxidants. (2017) 6:24. 10.3390/antiox6020024 28353636 PMC5488004

[6] Goff JP. Invited review: mineral absorption mechanisms, mineral interactions that affect acid-base and antioxidant status, and diet considerations to improve mineral status. J Dairy Sci. (2018) 101:2763–813. 10.3168/jds.2017-13112 29397180

[7] Owens B McCann MEE Preston C. The effect of substitution of inorganic zinc with proteinated or chelated zinc on broiler chick performance. J Appl Poult Res. (2009) 18:789–94. 10.3382/japr.2008-00122

[8] Liu FF Azad MAK Li ZH Li J Mo KB Ni HJ. Zinc supplementation forms influenced zinc absorption and accumulation in piglets. Animals. (2021) 11:36. 10.3390/ani11010036 33375418 PMC7824504

[9] Aliarabi H Fadayifar A Tabatabaci MM Zamani P Bahari A Farahavar A. Effect of zinc source on hematological, metabolic parameters and mineral balance in lambs. Biol Trace Elem Res. (2015) 168:82–90. 10.1007/s12011-015-0345-0 25910899

[10] European Commission (EC). Commission implementing regulation (EU) 2016/1095. Off J Eur Union. (2016) L 182:7–27.

[11] Cobanová K Chrastinová L Chrenková M Polačiková M Formelová Z Ivanišinová O. The effect of different dietary zinc sources on mineral deposition and antioxidant indices in rabbit tissues. World Rabbit Sci. (2018) 26:241–8. 10.4995/wrs.2018.9206

[12] Kosakowska O Baczek K Przybyl JL Pawełczak A Rolewska K Weglarz Z. Morphological and chemical traits as quality determinants of common thyme (*Thymus vulgaris* L.), on the example of 'standard winter' cultivar. Agronomy. (2020) 10:909. 10.3390/agronomy10060909

[13] Nieto G. A review on application and uses of *Thymus* in the food industry. Plants. (2020) 9:961. 10.3390/plants9080961 32751488 PMC7464319

[14] Salehi B Mishra AP Shukla I Sharifi-Rad M del Mar Contreras M Segura-Carretero A. Thymol, thyme, and other plant sources: health and potential uses. Phytother Res. (2018) 32:1688–706. 10.1002/ptr.6109 29785774

[15] Gonelimali FD Lin J Miao W Xuan J Charles F Chen M. Antimicrobial properties and mechanism of action of some plant extracts against food pathogens and spoilage microorganisms. Front Vet Sci. (2018) 9:1639. 10.3389/fmicb.2018.01639 30087662 PMC6066648

[16] Palmieri S Pellegrini M Ricci A Compagnone D Lo Sterzo C. Chemical composition and antioxidant activity of thyme, hemp and coriander extracts: a comparison study of maceration, soxhlet, UAE and RSLDE techniques. Foods. (2020) 9:1221. 10.3390/foods9091221 32887367 PMC7555591

[17] Dalle Zotte A Celia C Szendro Z. Herbs and spices inclusion as feedstuff or additive in growing rabbit diets and as additive in rabbit meat: a review. Livest Sci. (2016) 189:82–90. 10.1016/j.livsci.2016.04.024

[18] Dal Bosco A Gerencsér Z Szendro Z Mugnai C Cullere M Kovàcs M. Effect of dietary supplementation of spirulina (*Arthrospira platensis*) and thyme (*Thymus vulgaris*) on rabbit meat appearance, oxidative stability and fatty acid profile during retail display. Meat Sci. (2014) 96:114–9. 10.1016/j.meatsci.2013.06.021 23896145

[19] Placha I Chrastinova L Laukova A Cobanova K Takacova J Strompfova V. Effect of thyme oil on small intestine integrity and antioxidant status, phagocytic activity and gastrointestinal microbiota in rabbits. Acta Vet Hung. (2013) 61:197–208. 10.1556/avet.2013.012 23661388

[20] Association of Official Analytical Chemists. Official Methods of Analysis of AOAC International. Gaithesburg, MD, USA (2000).

[21] Van Soest PJ Robertson JB Lewis BA. Methods for dietary fiber, neutral detergent fiber, and nonstarch polysaccharides in relation to animal nutrition. J Dairy Sci. (1991) 74:3583–97. 10.3168/jds.S0022-0302(91)78551-2 1660498

[22] Chrastinová L Chrenková M Formelová Z Poláčiková M Cobanová K Lauková A. Effect of combinative dietary zinc supplementation and plant thyme extract on growth performance and nutrient digestibility in the diet for growing rabbits. Slovak J Anim Sci. (2018) 51:52–60.

[23] Petrič D Mravčáková D Kucková K Kišidayová S Cieslak A Szumacher-Strabel M. Impact of zinc and/or herbal mixture on ruminal fermentation, microbiota, and histopathology in lambs. Front Vet Sci. (2021) 8:660794. 10.3389/fvets.2021.630971 33681338 PMC7926268

[24] Taghouti M Martins-Gomes C Félix LM Schäfer J Santos JA Bunzel M. Polyphenol composition and biological activity of *Thymus citriodorus* and *Thymus vulgaris*: comparison with endemic Iberian thymus species. Food Chem. (2020) 331:127362. 10.1016/j.foodchem.2020.127362 32590268

[25] Pacifico S Piccolella S Papale F Nocera P Lettieri A Catauro M. A polyphenol complex from *Thymus vulgaris* L. plants cultivated in the Campania Region (Italy): new perspectives against neuroblastoma. J Funct Foods. (2016) 20:253–66. 10.1016/j.jff.2015.11.008

[26] Pisarčíková J Ocelová V Faix Š Plachá I Calderón AI. Identification and quantification of thymol metabolites in plasma, liver and duodenal wall of broiler chickens using UHPLC-ESI-QTOF-MS. Biomed Chromatogr. (2017) 31:1–12. 10.1002/bmc.3881 27808421

[27] Marklund S Marklund G. Involvement of the superoxide anion radical in the autoxidation of pyrogallol and a convenient assay for superoxide dismutase. Eur J Biochem. (1974) 47:469–74. 10.1111/j.1432-1033.1974.tb03714.x 4215654

[28] Paglia DE Valentine WN. Studies on the quantitative and qualitative characterization of erythrocyte glutathione peroxidase. J Lab Clin Med. (1967) 70:158–69. 6066618

[29] Aebi H. Catalase *in vitro*. Meth Enzymol. (1984) 105:121–6. 10.1016/S0076-6879(84)05016-3 6727660

[30] Benzie IFF Strain JJ. The ferric reducing ability of plasma (FRAP) as a measure of “antioxidant power”: the FRAP assay. Anal Biochem. (1996) 239:70–6. 10.1006/abio.1996.0292 8660627

[31] Jo C Ahn DU. Fluorometric analysis of 2-thiobarbituric acid reactive substances in Turkey. Poult Sci. (1998) 77:475–80. 10.1093/ps/77.3.475 9521463

[32] Bradford MM. A rapid and sensitive method for the quantitation of microgram quantities of protein utilizing the principle of protein-dye binding. Anal Biochem. (1976) 72:248–54. 10.1016/0003-2697(76)90527-3 942051

[33] Ellman GL. Tissue sulfhydryl groups. Arch Biochem Biophys. (1959) 82:70–7. 10.1016/0003-9861(59)90090-6 13650640

[34] Gresakova L Venglovska K Cobanova K. Dietary manganese source does not affect Mn, Zn and Cu tissue deposition and the activity of manganese-containing enzymes in lambs. J Trace Elem Med Biol. (2016) 38:138–43. 10.1016/j.jtemb.2016.05.003 27267351

[35] Fekete S Lebas F. Effect of a natural flavour (thyme extract) on the spontaneous feed ingestion, digestion coefficients and fattening parameters. Magy Allatorv Lapja. (1983) 38:121–5.

[36] Chrastinová L Cobanová K Chrenková M Poláčiková M Formelová Z Lauková A. Effect of dietary zinc supplementation on nutrient digestibility and fermentation characteristics of caecal content in physiological experiment with young rabbits. Slovak J Anim Sci. (2016) 49:23–31.

[37] Gerencsér Z Szendro Z Matics Z Radnai I Kovács M Nagy I. Effect of dietary supplementation of spirulina (*Arthrospira platensis*) and thyme (*Thymus vulgaris*) on apparent digestibility and productive performance of growing rabbits. World Rabbit Sci. (2014) 22:1–9. 10.4995/wrs.2014.1351

[38] Kandeil MA Mohamed AEH Gabbar MA Ahmed RR Ali SM. Ameliorative effects of oral ginger and/or thyme aqueous extract on productive and reproductive performance of V-line male rabbits. J Anim Physiol Anim Nutr. (2019) 103:1437–46. 10.1111/jpn.13147 31334576

[39] Abdel-Wareth AAA Metwally AE. Productive and physiological response of male rabbits to dietary supplementation with thyme essential oil. Animals. (2020) 10:1844. 10.3390/ani10101844 33050468 PMC7599667

[40] Alscher DM Braun N Biegger D Stuelten C Gawronski K Mürdter TE. Induction of metallothionein in proximal tubular cells by zinc and its potential as an endogenous antioxidant. Kidney Blood Press Res. (2005) 28:127–33. 10.1159/000084921 15812196

[41] Schanz M Schaaf L Dippon J Biegger D Fritz P Alscher MD. Renal effects of metallothionein induction by zinc *in vitro* and *in vivo*. BMC Nephrol. (2017) 18:91. 10.1186/s12882-017-0503-z 28302075 PMC5353879

[42] Sato M Mehra RK Bremner I. Measurement of plasma metallothionein-I in the assessment of the zinc status of zinc-deficient and stressed rats. J Nutr. (1984) 114:1683–89. 10.1093/jn/114.9.1683 6470825

[43] Ratliff BB Abdulmahdi W Pawar R Wolin MS. Oxidant mechanisms in renal injury and diseases. Antioxid Redox Signal. (2016) 25:119–45. 10.1089/ars.2016.6665 26906267 PMC4948213

[44] Gedikoglu A Sökmen M Çivit A. Evaluation of *Thymus vulgaris* and *Thymbra spicata* essential oils and plant extracts for chemical composition, antioxidant, and antimicrobial properties. Food Sci Nutr. (2019) 7:1704–14. 10.1002/fsn3.1007 31139383 PMC6526640

[45] Afonso AF Pereira OR Cardoso SM. Health-Promoting effects of *Thymus* phenolic-rich extracts: antioxidant, anti-inflammatory and antitumoral properties. Antioxidants. (2020) 9:814. 10.3390/antiox9090814 32882987 PMC7555682

[46] Soares JR Dinis TCP Cunha AP Almeida LM. Antioxidant activities of some extracts of *Thymus zygis*. Free Radic Res. (1997) 26:469–78. 10.3109/10715769709084484 9179593

[47] Hashemipour H Kermanshahi H Golian A Veldkamp T. Effect of thymol and carvacrol feed supplementation on performance, antioxidant enzyme activities, fatty acid composition, digestive enzyme activities, and immune response in broiler chickens. Poult Sci. (2013) 92:2059–69. 10.3382/ps.2012-02685 23873553

[48] Xu D Hu MJ Wang YQ Cui YL. Antioxidant activities of quercetin and its complexes for medicinal application. Molecules. (2019) 24:1123. 10.3390/molecules24061123 30901869 PMC6470739

[49] Yan JY Zhang GW Zhang C Tang L Kuang SY. Effect of dietary organic zinc sources on growth performance, incidence of diarrhoea, serum and tissue zinc concentrations, and intestinal morphology in growing rabbits. World Rabbit Sci. (2017) 25:43–9. 10.4995/wrs.2017.5770

[50] Cao J Henry PR Guo R Holwerda RA Toth JP Littell RC. Chemical characteristics and relative bioavailability of supplemental organic zinc sources for poultry and ruminants. J Anim Sci. (2000) 78:2039–54. 10.2527/2000.7882039x 10947086

[51] Liu SB Li SF Lu L Xie JJ Zhang LY Wang RL. The effectiveness of zinc proteinate for chicks fed a conventional corn-soybean meal diet. J Appl Poult Res. (2013) 22:396–403. 10.3382/japr.2012-00564

[52] King JC. Yet again, serum zinc concentrations are unrelated to zinc intakes. J Nutr. (2018) 148:1341–51. 10.1093/jn/nxy190 30184229

[53] Holodova M Cobanova K Sefcikova Z Barszcz M Tuśnio A Taciak M. Dietary zinc and fibre source can influence the mineral and antioxidant status of piglets. Animals. (2019) 9:497. 10.3390/ani9080497 31362348 PMC6720890

[54] Sandstead HH. Requirements and toxicity of essential trace elements, illustrated by zinc and copper. Am J Clin Nutr. (1995) 61:621s−4s. 10.1093/ajcn/61.3.621S 7879727

[55] Qiu J Lu X Ma L Hou C He J Liu B. Low-dose of organic trace minerals reduced fecal mineral excretion without compromising performance of laying hens. Asian Australas J Anim Sci. (2020) 33:588–96. 10.5713/ajas.19.0270 31480181 PMC7054597

[56] Tako E. Dietary plant-origin bio-active compounds, intestinal functionality, and microbiome. Nutrients. (2020) 12:3223. 10.3390/nu12113223 33105549 PMC7690256

[57] Bohn T. Dietary factors affecting polyphenol bioavailability. Nutr Rev. (2014) 72:429–52. 10.1111/nure.12114 24828476

