# Supplementary material for: Changes in the Antioxidant and Mineral Status of Rabbits After Administration of Dietary Zinc and/or Thyme Extract
Source: Front Vet Sci. 2021 Oct 22;8:740658. doi: 10.3389/fvets.2021.740658 (PMC8569448; doi:10.3389/fvets.2021.740658)
Supplement: Supplementary file 1 [file Table_1.DOCX]

**Supplementary TABLE S1** Analyzed mineral concentrations in the certified reference materials (CRM).

| Flame AAS (FAAS),  Zn mg/L , mg/kg DM | No. | Mean | True value | Recovery % | RSD % | |
| --- | --- | --- | --- | --- | --- | --- |
| Plasma ClinCheck, Level II, mg/L | 15 | 1.58±0.04 | 1.54 | 102.5 | 8.9 | |
| Bovine muscle ERM-BB184 | 15 | 131.4±0.57 | 146 | 93.7 | 1.67 | |
| Bovine liver BCR-185R | 15 | 136.4±1.78 | 138.6 | 98.3 | 2.21 | |
| Pig kidney ERM-BB186 | 15 | 130.7±1.93 | 134 | 97.5 | 5.69 | |
| Poultry feed LGC 7173 | 4 | 88.6±2.15 | 91 | 97.4 | 4.86 | |
| **Electrothermal AAS (ETAAS),**  **Mn µg/L, mg/kg DM** | **No.** | **Mean** | **True value** | **Recovery %** | **RSD %** | |
| Plasma ClinCheck, Level II, µg/L | 15 | 16.63±0.18 | 15.5 | 104.2 | 4.43 | |
| Chicken muscle NCS ZC73016 | 15 | 1.67±0.09 | 1.65 | 101.5 | | 21.11 |
| Bovine muscle ERM-BB184 | 15 | 0.287±0.01 | 0.276 | 104.1 | | 16.65 |
| **FAAS, mg Mn/kg DM** | **No.** | **Mean** | **True value** | **Recovery %** | | **RSD %** |
| Bovine liver BCR-185R | 15 | 10.74±0.88 | 11.07 | 97.1 | 17.16 | |
| Pig kidney ERM-BB186 | 15 | 7.07±0.10 | 7.26 | 97.4 | 5.69 | |
| Poultry feed LGC 7173 | 4 | 135.1±5.72 | 131 | 103.1 | 8.46 | |
| **FAAS, Cu mg/L, mg/kg DM** | **No.** | **Mean** | **True value** | **Recovery %** | **RSD %** | |
| Plasma ClinCheck, Level II, µg/L | 15 | 1.2±0.03 | 1.22 | 99.7 | 8.96 | |
| Bovine muscle ERM-BB184 | 15 | 2.48±0.21 | 2.31 | 104.0 | 33.13 | |
| Bovine liver BCR-185R | 15 | 267.8±2.20 | 277 | 97.4 | 3.24 | |
| Pig kidney ERM-BB186 | 15 | 35.7±0.25 | 36.5 | 97.8 | 2.76 | |
| Wheat NCS ZC73030 | 3 | 2.41±0.11 | 2.4 | 100.2 | 9.50 | |
| **FAAS, Fe mg/L, mg/kg DM** | **No.** | **Mean** | **True value** | **Recovery %** | **RSD %** | |
| Plasma ClinCheck, Level II, mg/L | 15 | 1.17±0.02 | 1.11 | 105.4 | 5.70 | |
| Bovine muscle ERM-BB184 | 15 | 72.7±1.78 | 75 | 97.6 | 9.51 | |
| Pig kidney ERM-BB186 | 15 | 247.9±3.10 | 255 | 97.2 | 11.80 | |
| Poultry feed LGC 7173 | 4 | 141.6±4.70 | 145 | 97.6 | 6.61 | |

No. – number of analyzed CRM samples, True value – certified values of CRM. FAAS – flame atomic absorption method, ETAAS – Electrothermal atomic absorption method. The accuracy of the analytical methods was determined by calculating recoveries (Recovery, %). The precision of the analytical methods was determined as the Relative Standard Deviation (RSD, %). Data presented as means ± SEM.

ClinCheck: Control of lyophilized human plasma (Recipe, Munich, Germany). Bovine liver BCR-185R, Bovine muscle ERM-BB184, Pig kidney ERM-BB186: Institute for Reference Materials and Measurements (IRMM, Geel, Belgium). Poultry feed LGC 7173: United Kingdom Accreditation Service (UKAS Reference Materials, UK). Wheat NCS ZC73030: China National Analysis Centre (Beijing, China).
